# From HIV to global health: opportunities and challenges

**DOI:** 10.7448/IAS.15.6.18068

**Published:** 2012-11-11

**Authors:** W El-Sadr

**Affiliations:** Columbia University and International Center for AIDS Care and Treatment Programs (ICAP), New York, USA

## Abstract

Remarkable advances have been achieved over the past decade in confronting the global HIV epidemic. By the end of 2010, 6.5 million persons living with HIV had initiated antiretroviral therapy in low and middle-income countries, the majority in sub Saharan Africa. Of the total of 2.5 new HIV infections that occurred in 2010, 1.9 million occurred in sub Saharan Africa. Nonetheless, 22 countries in sub Saharan Africa have experienced a decrease in HIV incidence. These remarkable achievements have involved a transformation of fragile health system. Examples include new models of care, task shifting, infrastructure enhancement, establishment of new data systems and mobilization of communities.

However, many of the same countries where HIV is prevalent also confront other health threats including high maternal and child mortality, high rates of tuberculosis and malaria as well as a burgeoning non-communicable chronic disease threat. Addressing these health threats requires taking the lessons learned from the HIV response and adapting them to confront these threats. Through building on the foundation established, similar progress may be achieved in addressing these health threats while maintaining the momentum of the HIV response.

